# Genomic analyses identify multiple Asian origins and deeply diverged mitochondrial clades in inbred brown rats (*Rattus norvegicus*)

**DOI:** 10.1111/eva.12572

**Published:** 2017-12-07

**Authors:** Emily E. Puckett, Olivia Micci‐Smith, Jason Munshi‐South

**Affiliations:** ^1^ Louis Calder Center – Biological Field Station Fordham University Armonk NY USA

**Keywords:** inbreeding, mitogenomes, mitonuclear discordance, rat strains

## Abstract

Over 500 strains of inbred brown rats (*Rattus norvegicus*) have been developed for use as a biomedical model organism. Most of these inbred lines were derived from the colony established at the Wistar Institute in 1906 or its descendants following worldwide distribution to research and breeding centers. The geographic source of the animals that founded the Wistar colony has been lost to history; thus, we compared 25 inbred rat strains to 326 wild rats from a global diversity dataset at 32 k SNPs, and 47 mitochondrial genomes to identify the source populations. We analyzed nuclear genomic data using principal component analyses and co‐ancestry heat maps, and mitogenomes using phylogenetic trees and networks. In the nuclear genome, inbred rats clustered together indicating a single geographic origin for the strains studied and showed admixed ancestral variation with wild rats in eastern Asia and western North America. The Sprague Dawley derived, Wistar derived, and Brown Norway strains each had mitogenomes from different clades which diverged between 13 and 139 kya. Thus, we posit that rats originally collected for captive breeding had high mitochondrial diversity that became fixed through genetic drift and/or artificial selection. Our results show that these important medical models share common genomic ancestry from a few source populations, and opportunities exist to create new strains with diverse genomic backgrounds to provide novel insight into the genomic basis of disease phenotypes.

## 1 INTRODUCTION

Brown rats (*Rattus norvegicus*) have been used as medical models for a diverse array of biomedical studies including those in physiology, neurology, behavior, nutrition, surgery, and toxicology. Experimentation on rats began by at least the 1850s with published studies from laboratories in France, Great Britain, and Germany (Lindsey & Baker, 2006). The earliest reports of rats used in US research laboratories can be traced to the Department of Neurology at the University of Chicago in the 1890s when a Swiss researcher, Adolf Meyer, introduced Henry Donaldson to albino rats as a research model. Donaldson's writings question whether the University of Chicago rat colony was from North American stock or imported from Europe (Lindsey & Baker, 2006). In 1906, Donaldson became the Scientific Director of the Wistar Institute in Philadelphia, USA; he brought four pairs of albino rats from the University of Chicago colony, and these were the presumed founders of the famed Wistar colony. In 1909, Helen Dean King began inbreeding the Wistar colony, and by 1920, there were two colonies (“inbred” and “outbred”) of Wistar rats. Many subsequent strains were derived from the outbred Wistar commercial stock (e.g., Lewis, Buffalo, Wistar Kyoto). Another set of strains were created by mating the outbred colony with other rats of unknown commercial or wild stock; for example, Long‐Evans was produced by breeding a male caught in Berkeley, USA, with a Wistar female, and Sprague Dawley was produced by breeding a hooded male with a white Douredoure female, a line assumed to contain Wistar ancestry (Lindsey & Baker, 2006). Finally, King produced the Brown Norway strain from wild rats caught in Philadelphia, USA (Lindsey & Baker, 2006). One complicating factor for understanding strain development at the Wistar Institute was the introduction of cottonseed meal into the rat diet in 1918 that resulted in death or low fertility in the Wistar colonies (Lindsey & Baker, 2006). To meet commercial demand for rats, the Institute purchased other commercial stock, yet this stock brought diseases that also resulted in increased mortality of the colony. Thus, not only did the Wistar colonies experience a bottleneck, but individuals of unknown origin were introduced in the early 1920s and these strains may have been Wistar rats from another facility. Not all rat strains have Wistar ancestry; for example, Maynie Rose Curtis produced several inbred lines including Fisher 344, Marshall 520, and August 7,322 from stocks she received from breeders, where the breeder's name became the name of the line. She also produced the Avon and Copenhagen lines that were named for cities in Connecticut, USA, and Denmark, respectively (Lindsey & Baker, 2006). Finally, the Fawn Hooded is an outbred stock originally produced by Norman Maier at the University of Michigan by crossing a German brown and a Lashley albino, the latter of which was from the laboratory of Karl Lashley of Harvard University (Hedrich, 2006). Contemporary Fawn Hooded strains may also be crossed with Long‐Evans depending on the breeding facility.

Despite the diverse number of strains available, the Wistar colonies had an outsized role in creating the diversity of inbred strains today. Approximately half of the greater than 500 strains have known Wistar ancestry (Aitman et al., 2008). Inbred lines are developed by brother–sister matings for more than 20 generations, often selecting which siblings to mate following screens for physiological or behavioral traits of interest (e.g., body size, hypertension, tameness). Once a strain is developed, animals are shipped to research institutes and/or medical supply companies where inbreeding continues; thus, genetic drift may occur within the same strain maintained at different facilities. A recent analysis of 29 inbred (sub)strains used whole genome sequencing (WGS) to identify selective sweeps at genes associated with the physiological traits selected in each line (Atanur et al., 2013).

Globally, brown rats form six evolutionary clusters with substructured populations in each background as evidenced by recent phylogeographic studies (Puckett et al., 2016; Song, Lan, & Kohn, 2014). Briefly, brown rats speciated in northern China and Mongolia where they evolved a commensal relationship with humans. Rats first expanded their range as humans developed agricultural settlements and later aboard overland transport and ships. Puckett et al. (2016) inferred five range expansions which explain the main axes of evolutionary clustering in rats (cluster names in italics throughout). The two earliest expansions were southward into *South‐East Asia* and eastward into modern Russia. The eastward expansion later extended to North America, with independent colonizations of the *Aleutian Archipelago* and the Pacific coast (e.g., *Western North America*). From South‐East Asia, rats expanded across Eurasia into *Western Europe*, then colonized *Northern Europe* (e.g., Fennoscandia, and sites in Central Europe including Germany and the Netherlands). The fifth range expansion moved rats aboard ships during the height of European colonialism in the 1600–1800s. Thus, the genomic signature in eastern North America, the Caribbean, South America, Africa, and Australasia are similar to *Western Europe* (Puckett et al., 2016).

The population genetic relationships between inbred strains, and between inbred stains and wild relatives (Song et al., 2014) have been investigated previously albeit with limited sampling of either the genome or the diversity of wild individuals. Early work to deduce relationships among strains used microsatellites, RAPDs, and isozymes (Canzian, 1997; Thomas, Chen, Jensen‐Seaman, Tonellato, & Twigger, 2003), while more recent work has investigated single nucleotide polymorphisms and variants (SNPs and SNVs, respectively; Atanur et al., 2013; Hermsen et al., 2015). Topological differences between phylogenetic trees and networks were observed across these different studies due to the (sub)strains genotyped, marker type, and analysis method. We do not propose to untangle the network of rat strains (STAR Consortium 2008), but instead place the strains within the geographic context of worldwide wild rat diversity. The history of the rat colony at the Wistar Institute suggests multiple putative origins of rats including countries in Western Europe where rat studies began in the mid‐1800s, Chicago, USA, and/or Philadelphia, USA. Strains such as the Copenhagen were known to be sourced from Denmark; thus, we hypothesize that inbred lines will share evolutionary similarity with multiple geographic locations.

## 2 METHODS

We used five pre‐existing datasets and sequenced 15 wild rats for our analyses. First, we used a dataset of 32k nuclear SNPs generated with ddRAD‐Seq and genotyped in 321 rats from around the globe (Dryad Digital Repository https://doi.org/10.5061/dryad.jb3tc; Puckett et al., 2016). To this dataset we added five rats from Chicago, USA, genotyped using the same ddRAD‐Seq approach (NCBI SRA: PRJNA344413). Second, we used WGS data from 33 individuals representing 25 inbred strains and seven substrains (NCBI SRA accessions: ERR224446‐ERR224468, ERR185960‐ERR185968; Table S1; Atanur et al., 2013; Baud et al., 2013) and a third dataset of 11 wild rats from Harbin, China (European Nucleotide Archive ERP001276; Deinum et al., 2015). Fourth, for mitogenome analyses we included wild rats caught in Copenhagen, Denmark (NCBI AJ428514), and Tokyo, Japan (NCBI DQ673917; Nilsson, Gullberg, Spotorno, Arnason, & Janke, 2003; Schlick et al., 2006). Fifth, we downloaded 39 *cytB* haplotypes (Abhyankar, Park, Tonolo, & Luthman, 2009; Balakirev & Rozhnov, 2012; Bastos et al., 2011; Lin et al., 2012; Lu et al., 2012; Pagès et al., 2010; Schlick et al., 2006; Song et al., 2014; Truong et al., 2009) with greater geographic coverage than the mitogenomes.

We selected ten individuals from our global collection of *R. norvegicus* samples for WGS, four from *Western Europe* (including two from continental Europe: England and France, and two from New York City, USA, to represent the expansion range), two within *South‐East Asia* (Philippines and Cambodia), *Northern Europe* (Sweden and Netherlands), and one sample each from the *Aleutian Islands* and *Western North America* (Table S1). Samples (4 ng RNase‐treated genomic DNA) were sequenced at the New York Genome Center on an Illumina HiSeq 2500 generating paired‐end reads. Average sequencing depth ranged from 24 to 38× per genome (NCBI SRA: PRJNA344413).

### 2.1 Nuclear genome analyses

We mapped reads for each individual within the inbred and Chinese rat genomes to the Rnor_6.0 reference genome (Gibbs et al., 2004) using Bowtie v2.2.6 (Langmead & Salzberg, 2012) with default parameters. We extracted the 32,127 SNPs that were called in the ddRAD‐Seq dataset using a position list with SAMTOOLS v1.2 mpileup function (Li et al., 2009). Using these data, we estimated genetic diversity (expected heterozygosity: H_E_, and mean number of alleles: A) in ARLEQUIN v3.5 (Excoffier & Lischer, 2010). We ran a principal component analysis (PCA) where we projected the inbred samples into the PC space from the global diversity dataset using EIGENSOFT v5.0.2 (Patterson, Price, & Reich, 2006; Price et al., 2006). We also investigated population structure using FINESTRUCTURE v2.0.7 (Lawson, Hellenthal, Myers, & Falush, 2012) on the 20 autosomal chromosomes (31,489 SNPs). We phased and imputed each chromosome using fastPHASE (Scheet & Stephens, 2006). In FINESTRUCTURE, we ran the unlinked model with 25% of the data used for initial EM estimation, 750,000 iterations of the MCMC with 50% used as burn‐in and 1,000 samples retained, 20,000 tree comparisons, and 500,000 steps for the tree maximization. We viewed MCMC trace files to confirm stability of all parameters.

### 2.2 Mitochondrial genome analyses

For samples with WGS data, we exported reads aligned to the mitochondrial genome using SAMTOOLS, then mapped those reads to a reference mitogenome (NCBI accession AY172581 which is a Brown Norway strain, BN/NHsdMcwi) in GENEIOUS v5.4 (http://www.geneious.com; Kearse et al., 2012) using default settings. We exported the consensus sequence from each assembly.

We analyzed the mitogenomes both as a network and phylogenetic tree. We aligned all 47 brown rat mitogenomes using MUSCLE (Edgar, 2004) within GENEIOUS, then built a NeighborNet network in SPLITSTREE v4.13.1 (Huson & Bryant, 2006). To understand divergence time between the brown rat clades, we downsampled each clade identified in the network to a single individual (*n* = 12). As phylogenetic software views polymorphisms as fixed substitutions between sequences, we downsampled to limit this influence on the estimation of the substitution rate, where an overestimate results in older divergence times. Haplotype selection may influence this rate, as individual haplotypes within a clade contain differing numbers of polymorphisms, thus selecting highly polymorphic haplotypes can overestimate divergence time. We selected mitogenome outgroups from *R. rattus* (NC_012374), *R. tanezumi* (EU273712), *R. exulans* (EU273711), and *Mus musculus* (NC_005089; Bayona‐Bafaluy et al., 2003; Robins et al., 2008), then aligned the genomes as above. Using the program BEAUTI, we set up a BEAST v1.8.0 (Drummond & Rambaut, 2007) input file with the following parameters: no partitioning of the data, a lognormal relaxed substitution model (Drummond, Ho, Phillips, & Rambaut, 2006), and a constant coalescent tree model (Kingman, 1982). We placed a fossil calibration (normal distribution, mean 11.8 Mya, std 0.5 Mya) on the root of the tree splitting *Mus* and *Rattus*; the settings were chosen so that 90% of the prior distribution was between 11 and 12.5 Mya (Benton & Donoghue, 2007; Robins et al., 2008). Within the CIPRES Science Gateway v3.3 (Miller, Pfeiffer, & Schwartz, 2010), we ran two independent iterations of BEAST for 10^8^ Markov chain Monte Carlo steps sampling every 10^4^ steps. For comparison, we ran a separate iteration where the input file contained the priors yet no sequence data. We observed that the independent runs converged and that the runs with data were better supported than the prior alone, using TRACER v1.6 (Rambaut & Drummond, 2009). We combined the independent runs following removal of 25% of MCMC steps as burn‐in using LOGCOMBINER v1.8, then visualized the tree with the highest median log credibility score using TREEANNOTATOR v1.8 and report node age and the 95% highest probability density (HPD). One branch of the consensus tree had a posterior probability of 0.58 (see below); thus, we ran DENSITREE v2.2.5 (Bouckaert & Heled, 2014) to observe alternative topologies.

To place our results within the context of previous work on brown rat mitochondrial diversity, we aligned 1,140 bp of *cytochrome‐B* (*cytB*) previously analyzed by Song et al. (2014), and extracted the same region from the wild and inbred mitogenomes. We screened for duplicate haplotypes using COLLAPSE v1.2 (Posada, 2004). We aligned data in GENEIOUS then built a NeighborNet network in SPLITSTREE. We named clades in this network when samples were concordant with our mitogenome results.

## 3 RESULTS

### 3.1 Nuclear genome analyses

Inbred rats had moderate genetic diversity measured as H_E_ and A (Table S2) when compared to sampling sites around the world. However, when individual lines were analyzed, inbred rats had the lowest genetic diversity of any population analyzed where H_E_ ranged from 0.005 to 0.039 and A 0.66–1.062. These results were consistent with expectations under inbreeding, where all lines taken together contained similar diversity to wild rats, but any individual strain had very low genetic diversity as strains were selected for different traits.

**Figure 1:**
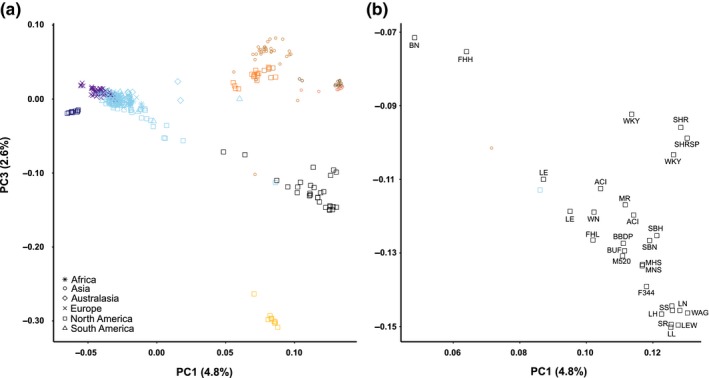
Principal component analyses of (a) the global diversity dataset (*n* = 326) of 32k SNPs and the inbred samples (*n* = 29; black) projected into the PC space for the first and third axes, (b) the inbred samples labeled (see Table S1) from the same projection. Sample colors indicate genomic clustering, including China (dark brown), South‐East Asia (light brown), eastern Russia (pink), Aleutian Archipelago (orange), Western North America (yellow), Northern Europe (purple), Western Europe and global expansion (light blue), and Haida Gwaii, Canada (dark blue)

**Figure 2:**
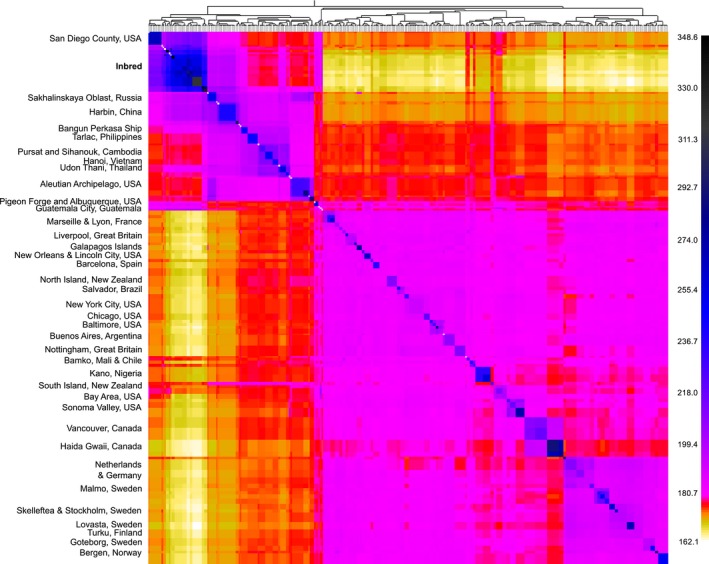
Co‐ancestry heat map of *Rattus norvegicus* (global diversity dataset *n* = 326; inbred *n* = 29) using 32k SNPs from the nuclear genome analyzed in FINESTRUCTURE, where yellow and black, respectively, denote lower and higher co‐ancestry

When inbred brown rats were projected into the PC space from a global diversity dataset, they clustered between samples from San Diego (i.e., *Western North America*), and eastern China and eastern Russia (Figure 1) on the third PC axis which distinguishes diversity in Asian samples. The first PC axis represents divergence between Asian and non‐Asian samples; inbred strains vary along this axis with Brown Norway showing the closest affinity to wild *Western Europe* rats (Figure 1). The results from FINESTRUCTURE were similar; first, the 25 strains formed a single cluster. When compared to the global diversity, inbred strains shared the most co‐ancestry with wild rats from the *Western North America* evolutionary cluster; co‐ancestry was moderately high with rats from eastern China and Russia (Figure 2). Inbred rats had distinctly low co‐ancestry values with European and eastern North American samples, except for samples from California, Tennessee, and New Mexico, USA, and Guatemala, which have an admixed signature between *Western Europe* and *Western North America* (Figure 2).

We observed 12 evolutionary clusters within the 29 inbred rats (Figure 1 and Figure S1). This method delineates population‐level substructure within the global dataset (Puckett et al., 2016) and likely picks up more closely shared ancestry within the inbred rat samples. Three samples including each of the Fawn Hooded strains (FHH/EurMcwi and FHL/EurMcWi) and Brown Norway formed their own cluster with a single sample (Figure S1). Unsurprisingly, substrains of the same strain also formed single clusters, including Long‐Evans, Wistar Kyoto, Spontaneously Hypertensive, Milan, and August x Copenhagen Irish. There were four clusters composed of varying backgrounds, including first, Lewis and Wistar Albino Glaxo; second, Lyon and Salt Sensitive/Resistant; third, Buffalo, Fisher 344, and Marshall 520; and fourth, Maudsley Reactive, Inbred Wistar, Sabra Hypertensive, and Biobreeding (Figure S1). We observed that one of the Fawn Hooded samples (FHH/EurMcwi), Long‐Evans, and Brown Norway clustered closer to samples from San Diego, USA, than all other strains.

### 3.2 Mitochondrial genome analyses

**Figure 3:**
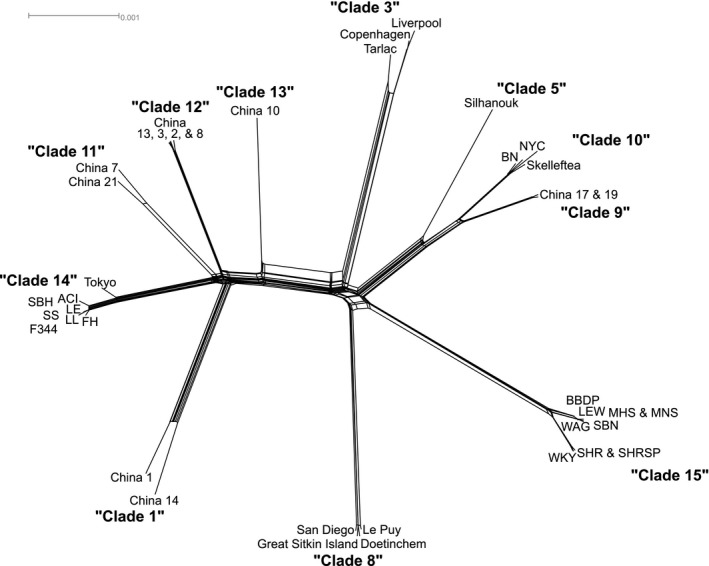
Network of *Rattus norvegicus* mitogenomes denoting either the geographic location of strain of wild and inbred rats, respectively (see Table S1). The name of each clade is listed in bold

We identified 11 clades within the 47 mitogenomes sequenced (Figure 3). The *cytB* network had similar patterns between the clades but with two additional clades not identified using the mitogenomes; additionally, clades 9 and 10 lacked sufficient resolution for differentiation (Figure S2). The inbred samples were distributed between clades 10, 14, and 15, a result that confirms earlier work (Schlick et al., 2006). The Brown Norway strain clustered with samples from Sweden and the USA, and was denoted as clade 10 by Puckett et al. (2016). Our *cytB* haplotype analysis identified haplotypes within this clade in China, France, Germany, Indonesia, and South Africa supporting previous results that this clade has a wide geographic distribution. Further, clade 10 was closely associated with the *Western Europe* evolutionary cluster that expanded globally, thus expanding its geographic reach. Long‐Evans, Fisher 344, Sabra Hypertension Prone, August x Copenhagen Irish, Fawn Hooded, Lyon, and Salt Sensitive/Resistant were in clade 14 with recent shared ancestry with a mitogenome sample from Tokyo, Japan (Figure 3), and *cytB* haplotypes from Germany and South Africa. Inbred Wistar, Wistar Kyoto, Wistar Albino Galaxo, Lewis, Milan, Sabra Hypertensive Resistant, and Spontaneously Hypertensive grouped into clade 15 that was not associated with any wild samples of known geographic origin. Substructure was apparent within clade 15 as strains from Wistar Kyoto and Spontaneously Hypertensive separated from other inbred lines (Figure 3). It was also notable that Puckett et al. (2016) underestimated the diversity from Harbin, China, originally grouping the 11 samples into two clades where the full mitogenome analysis identified six clades that we renamed clade 1, 9, 11–14 (Figure 3).

**Figure 4:**
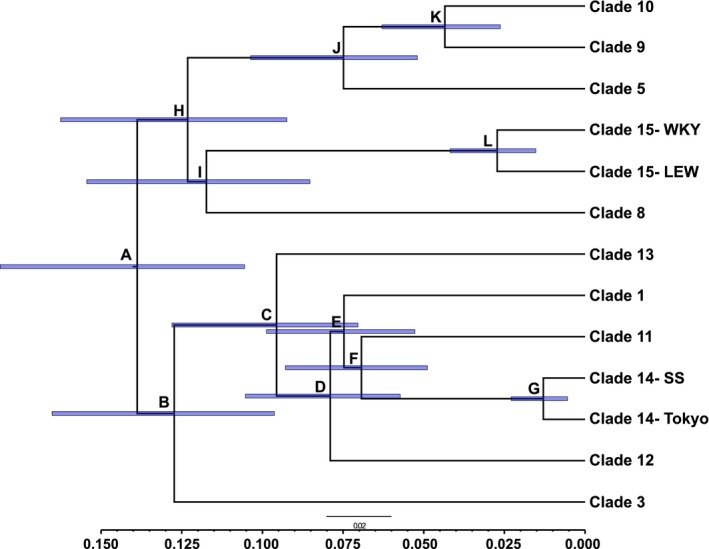
Phylogenetic tree of *Rattus norvegicus* mitochondrial genomes with *Rattus* and *Mus* outgroups removed for legibility (see Figure S3 for tree with outgroups). See Table 1 for posterior support, divergence times, and 95% HPD for each node

We estimated divergence time (Figure 4 and Figure S3) and mutation rate (0.023 substitutions per site per Ma; HPD 0.020–0.027) across the *Rattus* mitogenome tree. As expected, estimated divergence times between mice and rats, and within *Rattus* were similar to previously published results (Figure S3; Robins et al., 2008); thus, we focused on the timing of divergence within *R. norvegicus*. Notably, the Bayesian posterior probability for a sister relationship between clade 3 (node B) and other Asian samples was 0.58 (Table 1) where the DENSITREE analysis presents two alternative topologies for the placement of clade 3 (Figure S3). The *R. norvegicus* crown was 139 kya (HPD 105–181 kya; Figure 4, Table 1). Divergence times of other clades were primarily before the last glacial maximum (LGM; 18–22 kya), except for divergence of a sample from Tokyo, Japan, and the inbred strains in clade 14 (node G) where divergence was estimated following the last glacial maximum (Figure 4, Table 1).

**Table 1:** Divergence times with 95% highest posterior density (HPD) estimates and Bayesian posterior probabilities for each node in the *Rattus norvegicus* phylogenetic tree shown in Figure 4

Node	Divergence (kya)	HPD (kya)	Posterior
A	139	105–181	1.00
B	127	96–165	0.58
C	96	70–128	1.00
D	79	57–105	1.00
E	75	53–99	0.76
F	69	49–93	0.72
G	13	5–23	1.00
H	123	92–162	0.90
I	117	85–154	0.99
J	75	52–104	1.00
K	43	26–63	1.00
L	27	15–42	1.00

## 4 DISCUSSION

Within the 25 inbred rat strains that we investigated, the nuclear genomes formed a single genomic cluster of admixed Asian ancestry (Figure 2); thus, neither of our hypotheses were supported. We first hypothesized that inbred rats would cluster with *Western Europe* genotypes due to an assumption that colonies were founded by wild rats closest to the researchers in Europe and the USA that initially developed experimental colonies. The patterns of co‐ancestry suggest that the specific source population was not sampled in the global diversity dataset. Increased sampling throughout China, Russia, eastern Asia, and western North America may identify the source or alternatively show that the western North America, eastern Russia, and eastern China ancestry was admixed early during the development of inbred strains. The historic record for Brown Norway states this strain was derived from wild rats collected in Philadelphia, USA; however, this strain clusters with the other 24 lines. By extending the geographic extent of the data using the *cytB* network, we show the high prevalence of clade 10 haplotypes both in China and Europe, with additional geographic coverage in eastern North America, South America, Africa, and Australasia due to the global range expansion associated with the intense colonial period in Europe (Puckett et al., 2016). Brown Norway had higher co‐ancestry with *Western Europe* samples than other inbred strains. While this *Western Europe* ancestry and clade 10 mitogenome both suggest that rats from Philadelphia were included within the Brown Norway strain, the overall ancestry also suggests that much of the genome came from one of the Wistar colonies.

Our second hypothesis, that inbred rats would form multiple clusters due to independent domestication events, was not supported. We particularly expected to see this result within August × Copenhagen Irish where ancestry from Denmark, which is in the *Northern Europe* genomic cluster (Puckett et al., 2016), was expected. The lack of independent domestication events may indicate the early spread of individuals from the Wistar colony to other breeding facilities that were subsequently renamed and used in crosses. The STAR Consortium (2008) observed Wistar derived lines dispersed throughout their network, combined with our results of these inbred strains forming a single evolutionary cluster compared to global wild rat diversity, we must question the presumed lack of Wistar ancestry in lines not believed to be derived from Wistar rats. If there were multiple geographic origins of domestication, we would expect to observe inbred rats throughout our co‐ancestry heat map (Figure 2).

That inbred rats have moderately diverged mitogenomes was surprising given the nuclear results; however, several historical scenarios may explain the discordance. First, the samples from Harbin, China, had mitogenomes from five clades with divergence 69–139 kya (Figure 4, Table 1) yet a single nuclear genomic signature, thus suggesting ancient population structure and admixture not captured in the contemporary brown rat phylogeography. This pattern of maintaining diverse mitochondrial genomes as the signature of nuclear genome admixture homogenizes was also observed in an invasive population of *R. rattus* in western North America (Conroy et al., 2013). Second, the Pacific coast of North America has high mitochondrial diversity, including haplotypes belonging to clades 4, 8, and 10, and unsampled diversity may also be present (Lack, Hamilton, Braun, Mares, & Van Den Bussche, 2013; Puckett et al., 2016). Thus, it is likely that multiple mitochondrial clades were present in the original breeding population that inbreeding then fixed over time, or possibly in the case of Brown Norway was introduced into an inbred line through wild females.

The mitogenome phylogenetic tree had several interesting features. First, we estimated that the majority of mitogenome diversity was structured before the LGM except for diversity within clades 14 and 15 that diverged within glacial refugia or soon after glacial retreat (Figure 4). Both of these clades contain inbred samples and highlight that all of the natural mitochondrial variation has not been sampled from wild populations. Our inclusion of a geographically diverse *cytB* dataset supports this hypothesis of unsampled diversity around the globe as substructure increased with inclusion of more samples (Figure S2). Second, we note that our estimate of 139 kya (HPD 105–181 kya) for the brown rat crown was significantly less than the previous estimate of 1.33 Mya (HPD 436 kya–2.35 Mya; Song et al., 2014). Song et al. (2014) included all *cytB* haplotypes without downsampling the clades, thereby overestimating divergence time due to the inclusion of polymorphisms that would be viewed as fixed substitutions in a phylogenetic analysis. Both analyses contain haplotypes from clades covering the deepest split (Node A in Figure 4); thus, this was not the source of the different estimates. Third, our estimate of a substitution rate of 0.023 per site per Mya had little variation across the branches of the full tree and was lower than the 0.098 substitutions per third codon per Mya previously estimated for *R. norvegicus* (Nabholz, Glemin, & Galtier, 2008), yet our inclusion of all nucleotides in this estimate explains this difference. Fourth, we note an incongruence with the SNP haplotype network used to select samples for full mitogenome sequencing, including that the haplotype network underestimated divergence of the samples from Harbin, China, originally grouping them into two clades (clades 1 and 9) where the mitogenome network identified five clades with old divergence (Puckett et al., 2016).

Our results indicate that only a small portion of global genomic diversity has been captured within inbred rats, and current strains are most closely associated with the *Western North America* and *China* evolutionary clusters. Thus, there is substantial genomic variability in wild rats not accounted for in current medical models, although we acknowledge that we studied a subset of highly used strains in North American and European research laboratories; thus, there may be strains representing additional diversity. This finding parallels the skew in human genomewide association studies (GWAS), where linkage disequilibrium, private SNVs, allele frequencies, and genomic architecture differ between ancestral backgrounds, thus limiting the transferability of the highly studied Northern and Western European (CEU) population to other ancestral backgrounds and admixed populations (Bustamante, De La Vega, & Burchard, 2011; Need & Goldstein, 2009). Thus, the generation of new inbred rat lines from one or more backgrounds (e.g., *South‐East Asia* or *Western Europe*) may expand both the phenotypic diversity and our understanding of the genomic basis of disease (Chow, 2015). Developing and maintaining inbred lines is costly in both time and money, although such efforts may be rewarded by developing a broader understanding of the genomic architecture underlying traits with biomedical applications.

## DATA ARCHIVING STATEMENT

DNA sequence data for five ddRAD‐Seq samples and 10 WGS samples have been deposited in the NCBI Short Read Archive under BioProject PRJNA344413.
