# Supplementary material for: Genomic analyses identify multiple Asian origins and deeply diverged mitochondrial clades in inbred brown rats (Rattus norvegicus)
Source: Evol Appl. 2017 Dec 7;11(5):718–26. doi: 10.1111/eva.12572 (PMC5979757; doi:10.1111/eva.12572)
Supplement: Supplementary file 1 [file EVA-11-718-s001.docx]

**SUPPLEMENTAL INFORMATION**

**
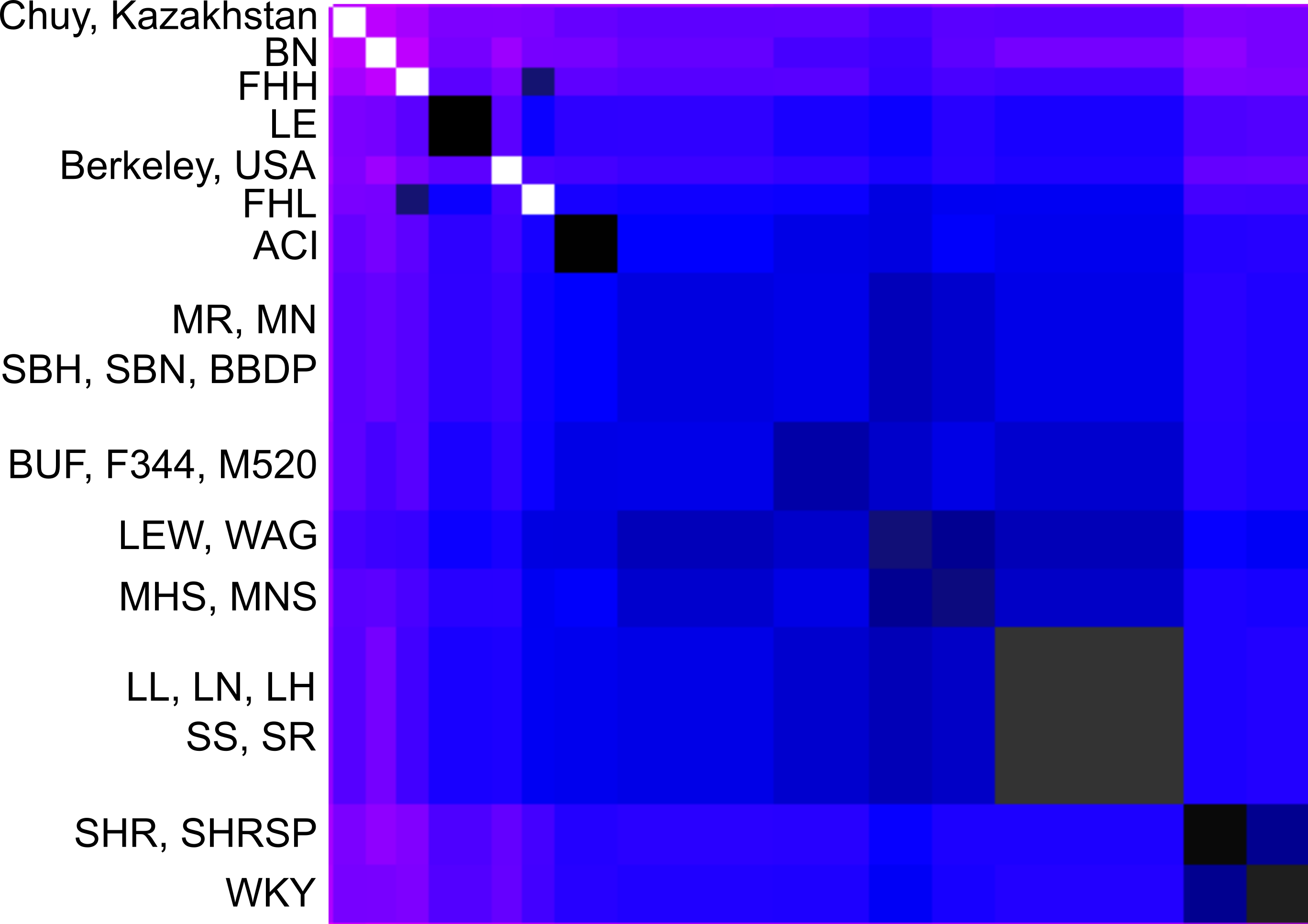
**

**Figure S1-** Zoomed in view of Figure 1 from main text highlighting the co-ancestry relationships between the inbred rats in this study. Inbred rats are listed by their strain names as in Table S1.


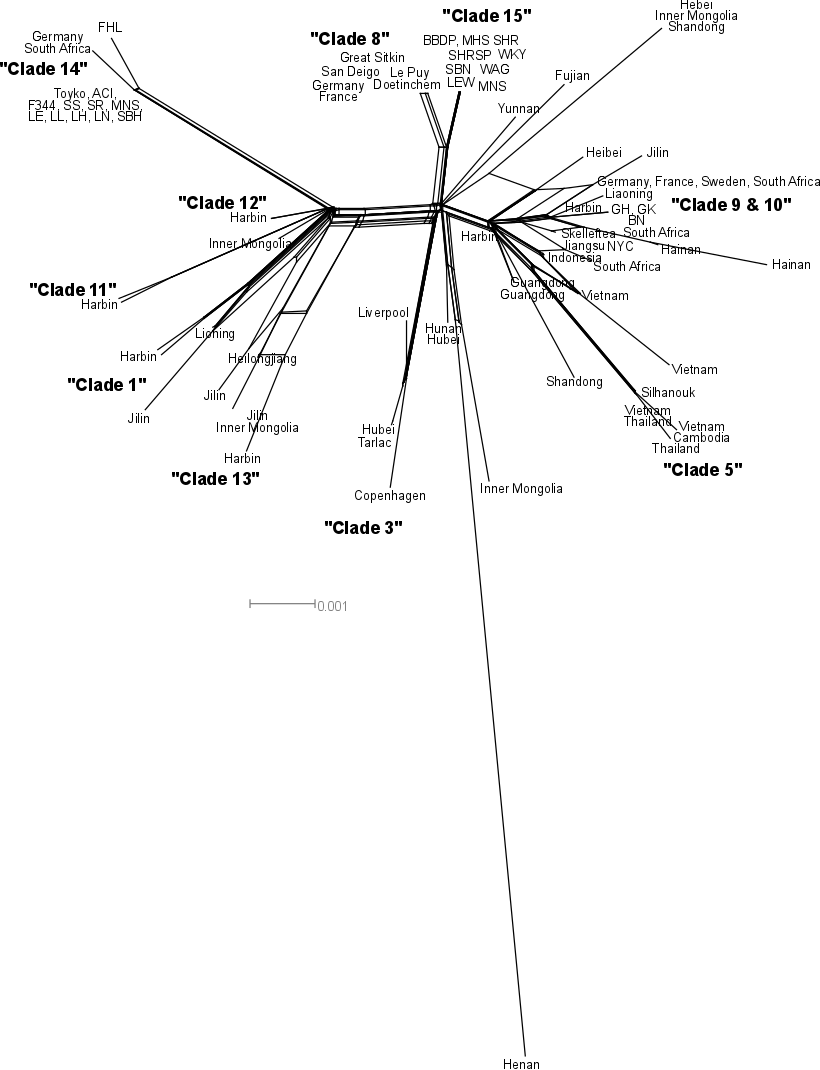


**Figure S2-** *Cytochrome-B* haplotype network (n = 45) with geographic location (city where available, country otherwise) or inbred stain name (see Table S1 for abbreviations). Clades were designated where divergence patterns matched the mitogenome results (Figure 3 of main text).


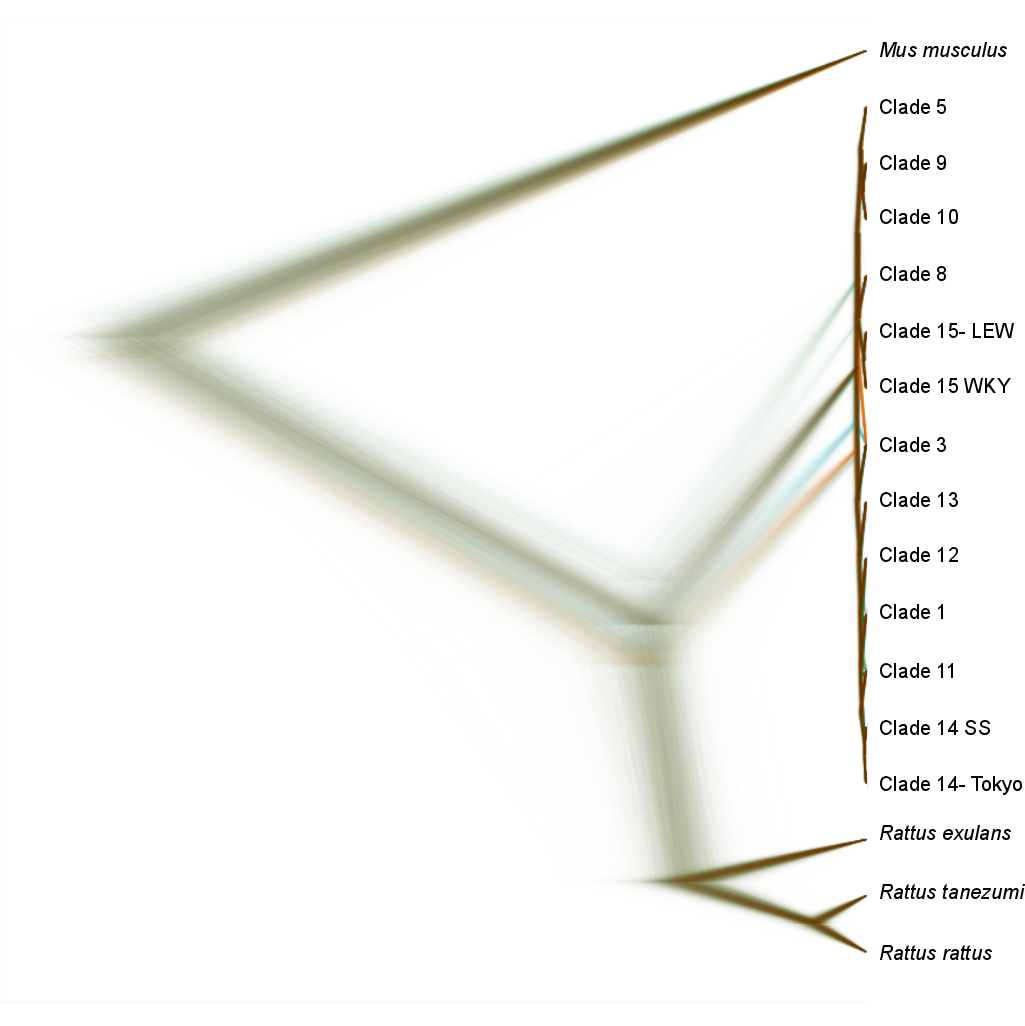


**Figure S3-** Full phylogenetic tree with *Mus* and *Rattus* outgroups (italicized) and detailing alternative topologies for the placement of clades within *R. norvegicus* (see Figure 4 in main text for detailed topology of highest clade creditability tree).

**Table S1-** List of samples used in this study for both inbred and wild individuals with substrain or geographic location, respectively. Accessions can be found in the respective NCBI (SRA or GenBank) or ENA database. We distinguish if the mitochondrial and/or nuclear genome was used in our analyses as quality of the accessions varied between samples.

|  |  |  |  |  |  |  |  |
| --- | --- | --- | --- | --- | --- | --- | --- |
| **Sample Name** | | **Strain or Geographic Location** | **Database** | **Accession** | **Source** | **Mito** | **Nuclear** |
| ***Inbred*** | |  |  |  |  |  |  |
|  | ACI/EurMcwi | August × Copenhagen Irish | SRA | ERR224446 | Atanur et al 2013 | X | X |
|  | ACI/N | August × Copenhagen Irish | SRA | ERR185960 | Baud *et al*. 2014 |  | X |
|  | BBDP/Wor | Biobreeding- Diabetes Prone | SRA | ERR224447 | Atanur et al 2013 | X | X |
|  | BN/NHsdMcwi | Brown Norway | GenBank | AY172581 | Gibbs *et al*. 2004 | X |  |
|  | Bn/SsN | Brown Norway | SRA | ERR185961 | Baud *et al*. 2014 |  | X |
|  | BUF/N | Buffalo | SRA | ERR185962 | Baud *et al*. 2014 |  | X |
|  | F344/N | Fischer 344 | SRA | ERR185963 | Baud *et al*. 2014 |  | X |
|  | F344/NCrl | Fischer 344 | SRA | ERR224448 | Atanur et al 2013 | X |  |
|  | FHH/EurMcwi | Fawn Hooded- High Blood Pressure | SRA | ERR224449 | Atanur et al 2013 | X | X |
|  | FHL/EurMcwi | Fawn Hooded- Low Blood Pressure | SRA | ERR224450 | Atanur et al 2013 | X | X |
|  | LE/Stm | Long Evans | SRA | ERR224452 | Atanur et al 2013 | X | X |
|  | LE/Stm | Long Evans | SRA | ERR185968 | Baud *et al.* 2014 |  | X |
|  | Lew/Crl | Lewis | SRA | ERR224453 | Atanur et al 2013 | X | X |
|  | Lew/NCrlBR | Lewis | SRA | ERR224454 | Atanur et al 2013 | X |  |
|  | LH/MavRrrc | Lyon Hypertensive | SRA | ERR224455 | Atanur et al 2013 | X | X |
|  | LL/MavRrrc | Lyon Hypotensive | SRA | ERR224457 | Atanur et al 2013 | X | X |
|  | LN/MavRrrc | Lyon Normotensive | SRA | ERR224456 | Atanur et al 2013 | X | X |
|  | M520/N | Marshall 520 | SRA | ERR185964 | Baud *et al*. 2014 |  | X |
|  | MHS/Gib | Milan Hypertensive Strain | SRA | ERR224458 | Atanur et al 2013 | X | X |
|  | MNS/Gib | Milan Normotensive Strain | SRA | ERR224459 | Atanur et al 2013 | X | X |
|  | MR/N | Maudsely Reactive | SRA | ERR185965 | Baud *et al*. 2014 |  | X |
|  | SBH/Ygl | Sabra Hypertension Prone | SRA | ERR224460 | Atanur et al 2013 | X | X |
|  | SBN/Ygl | Sabra Hypertension Resistant | SRA | ERR224461 | Atanur et al 2013 | X | X |
|  | SHR/NHsd | Spontaneously Hypertensive | SRA | ERR224462 | Atanur et al 2013 | X | X |
|  | SHRSP/Gla | Spontaneously Hypertensive-Stroke Prone | SRA | ERR224463 | Atanur et al 2013 | X | X |
|  | SR/Jr | Salt Resistant | SRA | ERR224464 | Atanur et al 2013 | X | X |
|  | SS/Jr | Salt Sensitive | SRA | ERR224465 | Atanur et al 2013 | X | X |
|  | SS/JrHsdMcwi | Salt Sensitive | SRA | ERR224466 | Atanur et al 2013 | X | X |
|  | WAG/Rij | Wistar Albino Glaxo | SRA | ERR224467 | Atanur et al 2013 | X | X |
|  | WKY/N | Wistar Kyoto | SRA | ERR185966 | Baud *et al*. 2014 |  | X |
|  | WKY/NCrl | Wistar Kyoto | SRA | ERR224468 | Atanur et al 2013 | X | X |
|  | WKY/NHsd | Wistar Kyoto | SRA | ERR224470 | Atanur et al 2013 | X |  |
|  | WN/N | Inbred Wistar | SRA | ERR185967 | Baud *et al*. 2014 |  | X |
| ***Wild Type*** | |  |  |  |  |  |  |
|  | FRA18 | LePuy, France | SRA | PRJNA344413 | This study | X | X |
|  | GBR11 | Liverpool, England | SRA | PRJNA344413 | This study | X | X |
|  | KHM07 | Silhanouk, Cambodia | SRA | PRJNA344413 | This study | X | X |
|  | NLD21 | Doetinchem, Netherlands | SRA | PRJNA344413 | This study | X | X |
|  | PHL09 | Tarlac, Philippines | SRA | PRJNA344413 | This study | X | X |
|  | SWE35 | Skelleftea, Sweden | SRA | PRJNA344413 | This study | X | X |
|  | Rn246 | New York City, USA | SRA | PRJNA344413 | This study | X | X |
|  | Rn255 | New York City, USA | SRA | PRJNA344413 | This study | X | X |
|  | ALK20 | Great Sitkin Island, USA | SRA | PRJNA344413 | This study | X | X |
|  | CAL79 | San Diego, USA | SRA | PRJNA344413 | This study | X | X |
|  | Copenhagen | Copenhagen, Denmark | GenBank | AJ428514 | Nilsson *et al.* 2003 | X |  |
|  | Tokyo | Tokyo, Japan | GenBank | DQ673917 | Schlick *et al.* 2006 | X |  |
|  | China1 | Harbin, China | ENA | ERP001276 | Deinum *et al.* 2015 | X | X |
|  | China2 | Harbin, China | ENA | ERP001276 | Deinum *et al.* 2015 | X | X |
|  | China3 | Harbin, China | ENA | ERP001276 | Deinum *et al.* 2015 | X | X |
|  | China7 | Harbin, China | ENA | ERP001276 | Deinum *et al.* 2015 | X | X |
|  | China8 | Harbin, China | ENA | ERP001276 | Deinum *et al.* 2015 | X | X |
|  | China10 | Harbin, China | ENA | ERP001276 | Deinum *et al.* 2015 | X | X |
|  | China13 | Harbin, China | ENA | ERP001276 | Deinum *et al.* 2015 | X | X |
|  | China14 | Harbin, China | ENA | ERP001276 | Deinum *et al.* 2015 | X | X |
|  | China17 | Harbin, China | ENA | ERP001276 | Deinum *et al.* 2015 | X | X |
|  | China19 | Harbin, China | ENA | ERP001276 | Deinum *et al.* 2015 | X | X |
|  | China21 | Harbin, China | ENA | ERP001276 | Deinum *et al.* 2015 | X | X |

**Table S2-** Summary statistics for genetic diversity estimates including mean and standard deviation (s.d.) expected heterozygosity (H_E_) and number of alleles (A) by country.

|  |  |  |  |  |  |  |
| --- | --- | --- | --- | --- | --- | --- |
| **Genomic Cluster** | **Location** | **Sample Size** | **H_E_** | **H_E_ s.d.** | **A** | **A s.d.** |
| **Inbred** |  |  |  |  |  |  |
|  | Inbred- All | 31 | 0.178 | 0.184 | 1.632 | 0.482 |
|  | ACI | 2 | 0.017 | 0.097 | 1.017 | 0.210 |
|  | BBDP | 1 | 0.013 | 0.114 | 0.996 | 0.174 |
|  | BN | 1 | 0.005 | 0.072 | 0.962 | 0.216 |
|  | BUF | 1 | 0.024 | 0.154 | 0.980 | 0.262 |
|  | FHH | 1 | 0.005 | 0.073 | 0.660 | 0.485 |
|  | FHL | 1 | 0.007 | 0.081 | 0.992 | 0.146 |
|  | F344 | 2 | 0.024 | 0.140 | 0.994 | 0.260 |
|  | LE | 2 | 0.019 | 0.103 | 1.028 | 0.205 |
|  | Lew | 2 | 0.008 | 0.086 | 0.995 | 0.153 |
|  | LH | 1 | 0.007 | 0.082 | 0.992 | 0.147 |
|  | LL | 1 | 0.007 | 0.082 | 0.991 | 0.151 |
|  | LN | 1 | 0.007 | 0.082 | 0.991 | 0.150 |
|  | M520 | 1 | 0.025 | 0.156 | 0.980 | 0.263 |
|  | MHS | 1 | 0.007 | 0.083 | 0.991 | 0.150 |
|  | MNS | 1 | 0.009 | 0.097 | 0.994 | 0.158 |
|  | MR | 1 | 0.031 | 0.173 | 0.986 | 0.274 |
|  | SBH | 1 | 0.007 | 0.086 | 0.992 | 0.152 |
|  | SBN | 1 | 0.011 | 0.102 | 0.993 | 0.167 |
|  | SHR | 1 | 0.008 | 0.091 | 0.991 | 0.161 |
|  | SHRSP | 1 | 0.006 | 0.079 | 0.991 | 0.146 |
|  | SR | 1 | 0.007 | 0.082 | 0.991 | 0.150 |
|  | SS | 2 | 0.015 | 0.100 | 1.016 | 0.178 |
|  | WAG | 1 | 0.008 | 0.090 | 0.993 | 0.154 |
|  | WKY | 2 | 0.039 | 0.145 | 1.062 | 0.270 |
|  | WN | 1 | 0.020 | 0.141 | 0.976 | 0.254 |
| **China** |  |  |  |  |  |  |
|  | China | 14 | 0.219 | 0.195 | 1.694 | 0.461 |
|  | Eastern Russia | 7 | 0.213 | 0.212 | 1.574 | 0.503 |
| **Southeast Asia** |  |  |  |  |  |  |
|  | Cambodia | 9 | 0.216 | 0.206 | 1.607 | 0.509 |
|  | Vietnam | 5 | 0.211 | 0.224 | 1.511 | 0.517 |
|  | Japan | 1 | 0.206 | 0.404 | 1.184 | 0.441 |
|  | Thailand | 9 | 0.235 | 0.202 | 1.683 | 0.477 |
|  | Philippines | 7 | 0.195 | 0.211 | 1.527 | 0.509 |
|  | Kyrgyzstan | 1 | 0.181 | 0.385 | 1.154 | 0.429 |
| **Aleutians** |  |  |  |  |  |  |
|  | Alaska, USA | 23 | 0.269 | 0.177 | 1.864 | 0.352 |
| **Western North America** | |  |  |  |  |  |
|  | San Diego, USA | 9 | 0.160 | 0.203 | 1.451 | 0.506 |
| **Northern Europe** |  |  |  |  |  |  |
|  | Norway | 8 | 0.178 | 0.214 | 1.465 | 0.510 |
|  | German | 7 | 0.246 | 0.208 | 1.672 | 0.479 |
|  | Netherlands | 14 | 0.258 | 0.188 | 1.808 | 0.403 |
|  | Sweden | 35 | 0.270 | 0.172 | 1.899 | 0.312 |
|  | Finland | 5 | 0.262 | 0.215 | 1.662 | 0.482 |
| **Western Europe** |  |  |  |  |  |  |
|  | Great Britain | 14 | 0.275 | 0.181 | 1.840 | 0.377 |
|  | France | 15 | 0.268 | 0.184 | 1.827 | 0.397 |
|  | Spain | 5 | 0.227 | 0.226 | 1.554 | 0.508 |
|  | Central Europe | 2 | 0.224 | 0.283 | 1.382 | 0.517 |
| **Western Europe- Expansion** | |  |  |  |  |  |
|  | Mali | 4 | 0.222 | 0.232 | 1.509 | 0.523 |
|  | Nigeria | 10 | 0.195 | 0.213 | 1.516 | 0.517 |
|  | Senegal | 1 | 0.116 | 0.320 | 1.085 | 0.374 |
|  | Argentina | 9 | 0.265 | 0.196 | 1.753 | 0.442 |
|  | Barbados | 3 | 0.228 | 0.246 | 1.482 | 0.518 |
|  | Brazil | 6 | 0.255 | 0.210 | 1.672 | 0.487 |
|  | Chile | 4 | 0.259 | 0.227 | 1.606 | 0.510 |
|  | Ecuador | 3 | 0.127 | 0.216 | 1.263 | 0.464 |
|  | Guatemala | 1 | 0.203 | 0.402 | 1.177 | 0.445 |
|  | Panama | 2 | 0.214 | 0.276 | 1.372 | 0.508 |
|  | Saint Lucia | 1 | 0.338 | 0.473 | 1.320 | 0.504 |
|  | Baltimore, USA | 9 | 0.266 | 0.194 | 1.767 | 0.433 |
|  | Chicago, USA | 4 | 0.206 | 0.232 | 1.474 | 0.519 |
|  | Lincoln City, USA | 4 | 0.164 | 0.228 | 1.356 | 0.501 |
|  | New Orleans, USA | 3 | 0.254 | 0.248 | 1.534 | 0.517 |
|  | NYC, USA | 10 | 0.281 | 0.186 | 1.817 | 0.396 |
|  | Pigeon Forge, USA | 1 | 0.224 | 0.417 | 1.203 | 0.453 |
|  | Albuquerque, USA | 3 | 0.183 | 0.235 | 1.393 | 0.506 |
|  | Bay Area, USA | 11 | 0.274 | 0.187 | 1.806 | 0.413 |
|  | Sonoma Valley, USA | 12 | 0.227 | 0.196 | 1.704 | 0.465 |
|  | Haida Gwaii, Canada | 11 | 0.121 | 0.179 | 1.396 | 0.500 |
|  | Vancouver, Canada | 15 | 0.246 | 0.195 | 1.742 | 0.446 |
|  | New Zealand | 9 | 0.270 | 0.195 | 1.771 | 0.430 |
